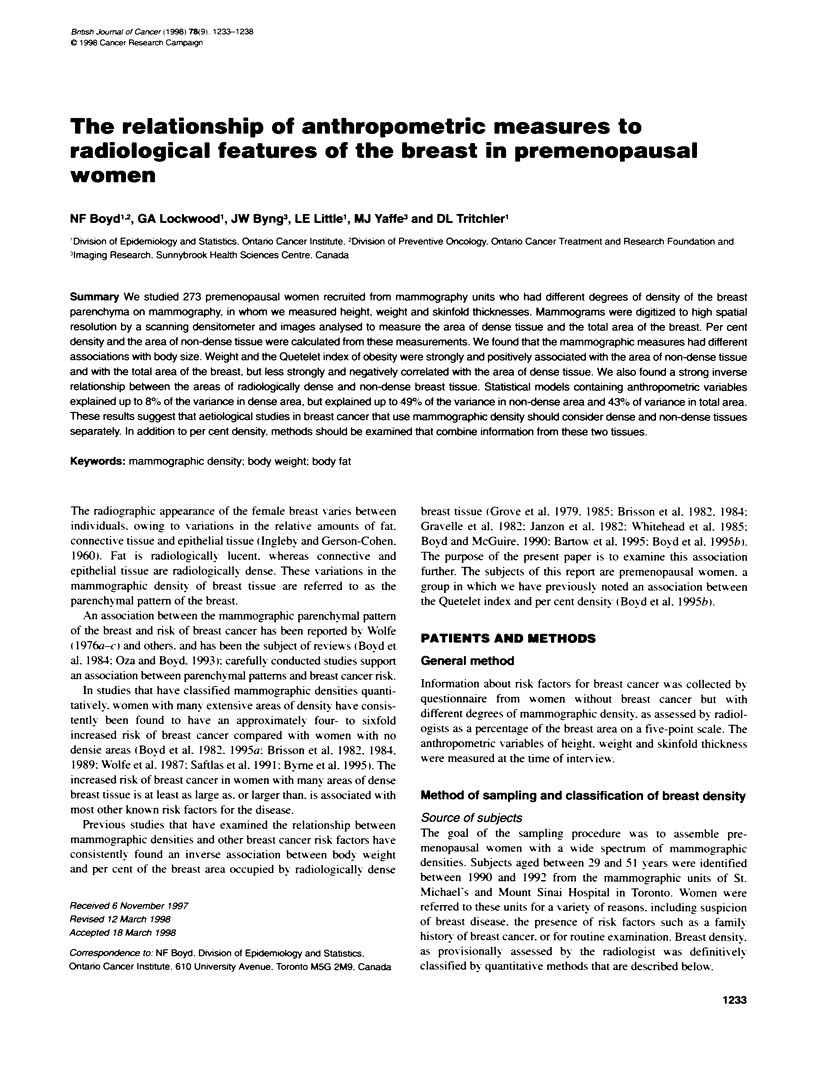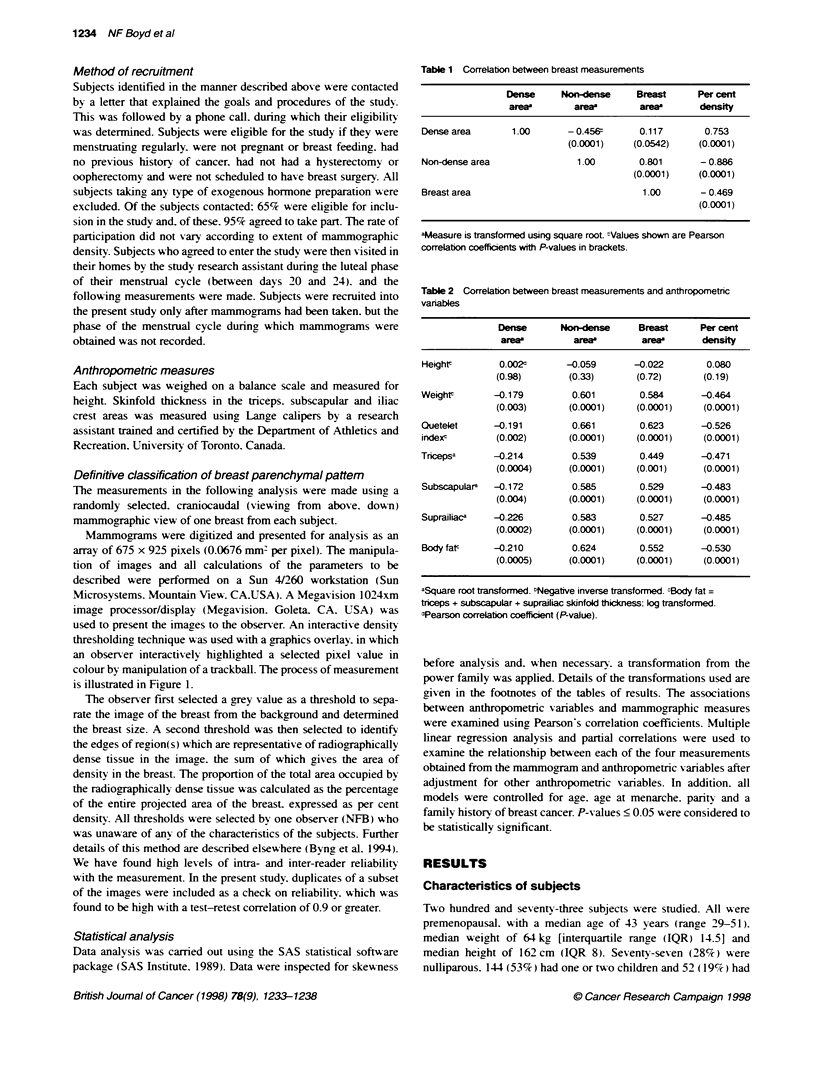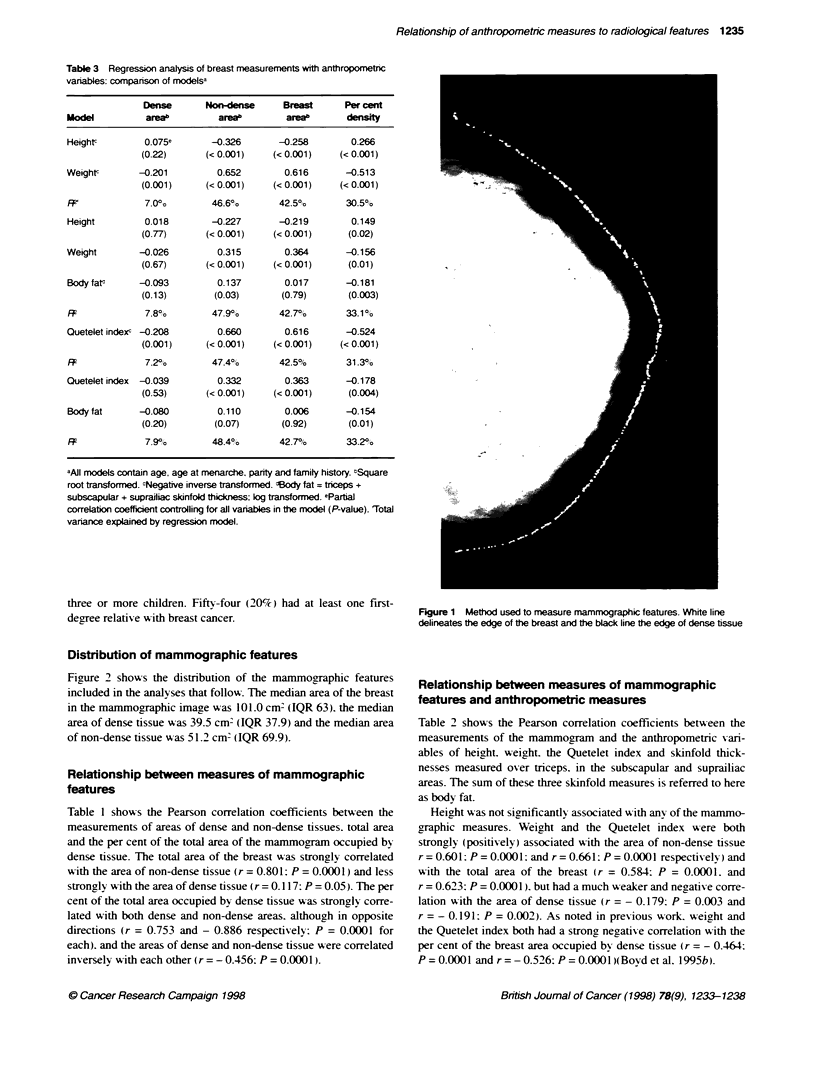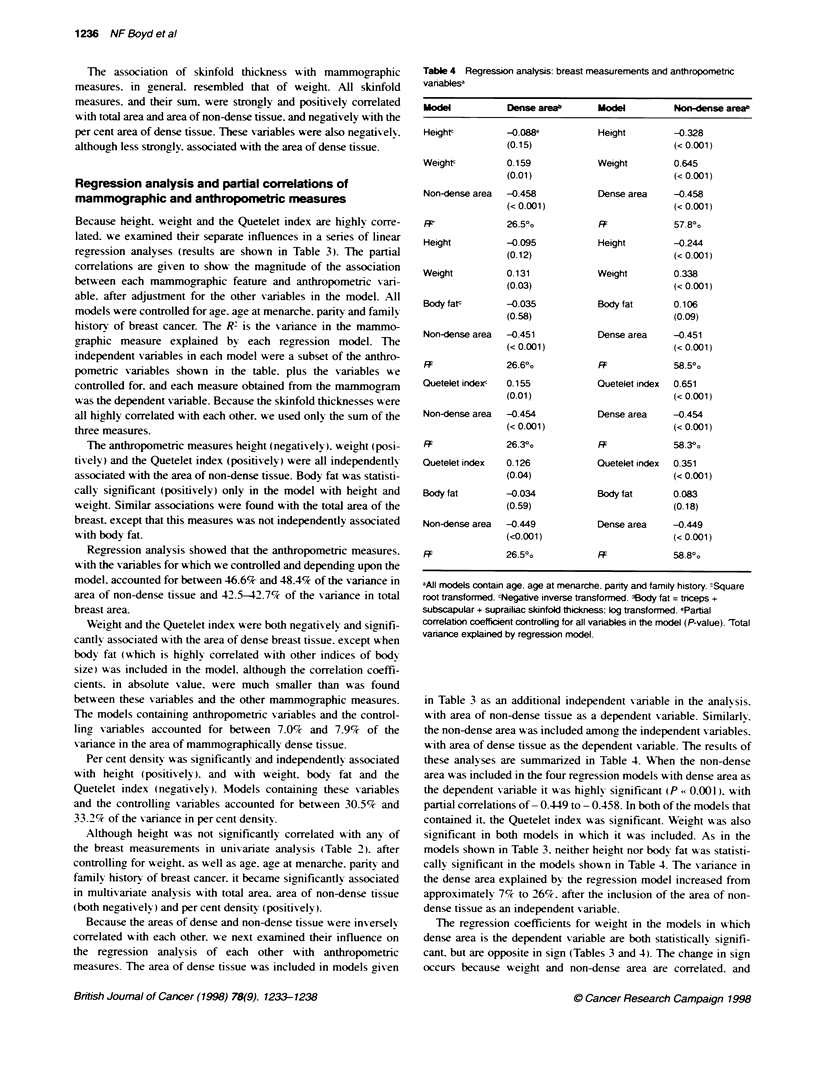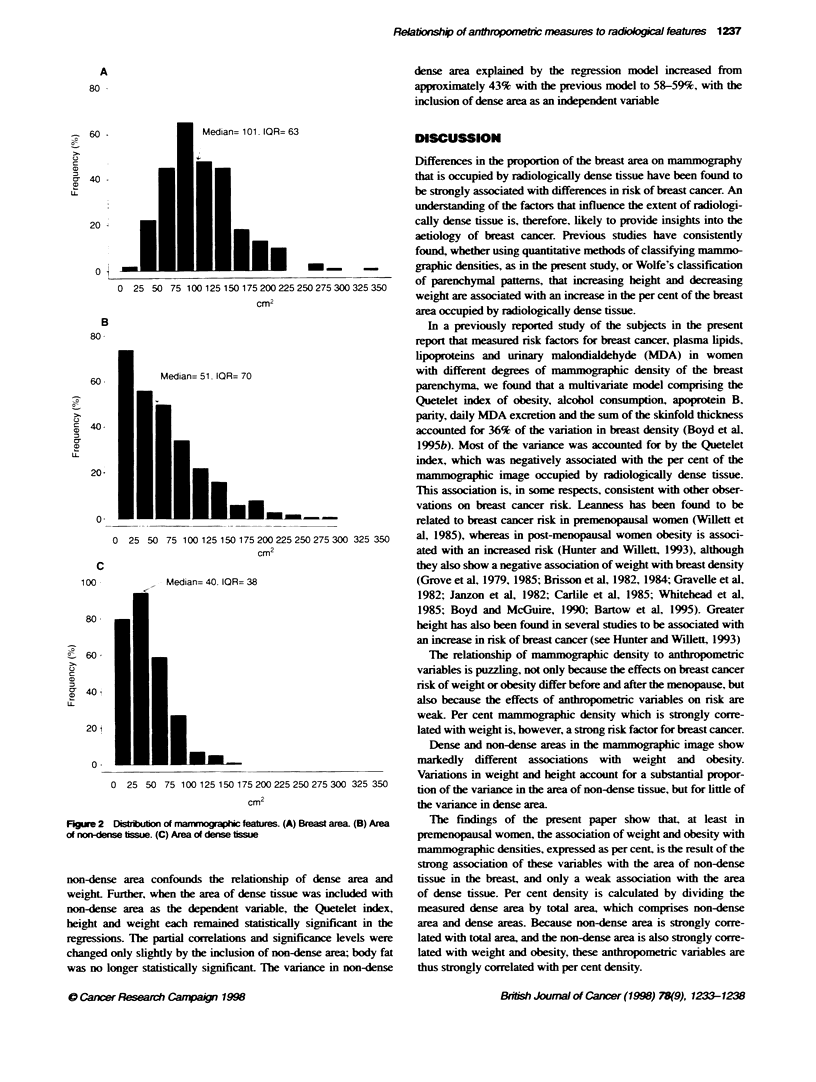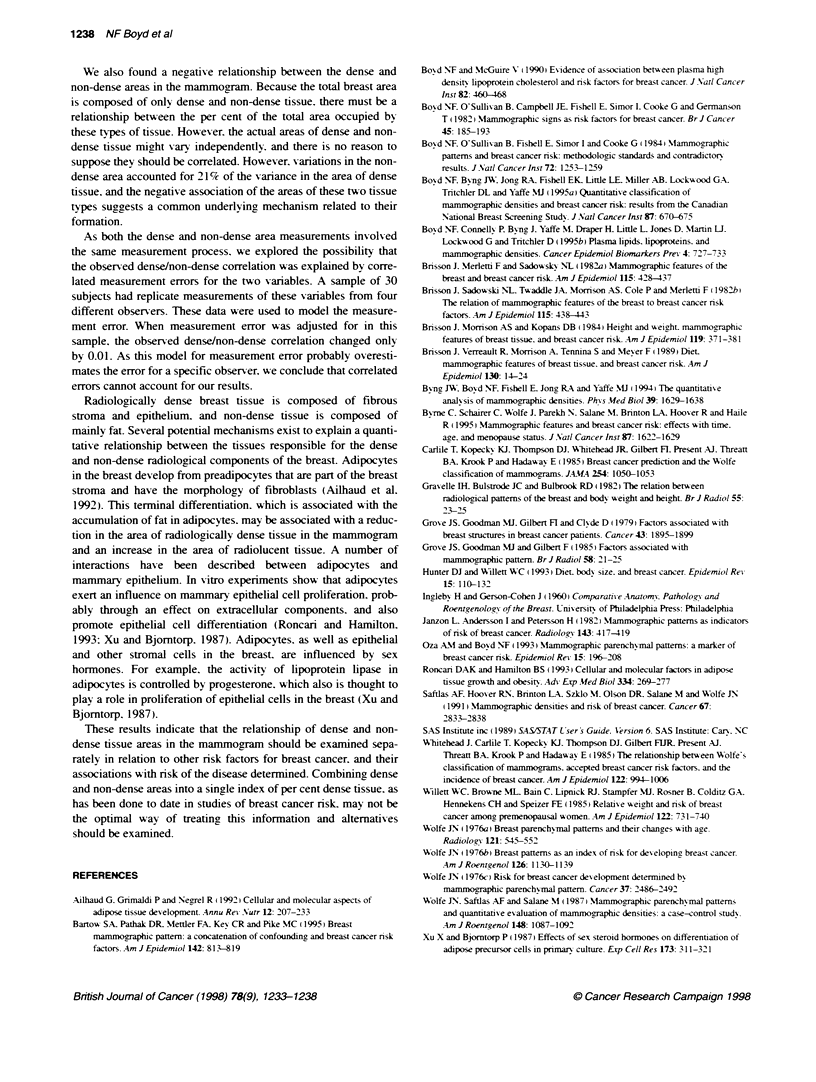# The relationship of anthropometric measures to radiological features of the breast in premenopausal women.

**DOI:** 10.1038/bjc.1998.660

**Published:** 1998-11

**Authors:** N. F. Boyd, G. A. Lockwood, J. W. Byng, L. E. Little, M. J. Yaffe, D. L. Tritchler

**Affiliations:** Division of Epidemiology and Statistics, Ontario Cancer Institute, Toronto, Canada.

## Abstract

**Images:**


British Journal of Cancer (1998) 78(9), 1233–1238
© 1998 Cancer Research Campaign

### The relationship of anthropometric measures to radiological features of the breast in premenopausal women

NF Boyd[1,2], GA Lockwood[1], JW Byng[3], LE Little[1], MJ Yaffe[3] and DL Tritchler[1]

[1]Division of Epidemiology and Statistics, Ontario Cancer Institute, [2]Division of Preventive Oncology, Ontario Cancer Treatment and Research Foundation and [3]Imaging Research, Sunnybrook Health Sciences Centre, Canada

**Summary** We studied 273 premenopausal women recruited from mammography units who had different degrees of density of the breast parenchyma on mammography, in whom we measured height, weight and skinfold thicknesses. Mammograms were digitized to high spatial resolution by a scanning densitometer and images analysed to measure the area of dense tissue and the total area of the breast. Per cent density and the area of non-dense tissue were calculated from these measurements. We found that the mammographic measures had different associations with body size. Weight and the Quetelet index of obesity were strongly and positively associated with the area of non-dense tissue and with the total area of the breast, but less strongly and negatively correlated with the area of dense tissue. We also found a strong inverse relationship between the areas of radiologically dense and non-dense breast tissue. Statistical models containing anthropometric variables explained up to 8% of the variance in dense area, but explained up to 49% of the variance in non-dense area and 43% of variance in total area. These results suggest that aetiological studies in breast cancer that use mammographic density should consider dense and non-dense tissues separately. In addition to per cent density, methods should be examined that combine information from these two tissues.

**Keywords:** mammographic density; body weight; body fat

The radiographic appearance of the female breast varies between individuals, owing to variations in the relative amounts of fat, connective tissue and epithelial tissue (Ingleby and Gerson-Cohen, 1960). Fat is radiologically lucent, whereas connective and epithelial tissue are radiologically dense. These variations in the mammographic density of breast tissue are referred to as the parenchymal pattern of the breast.

An association between the mammographic parenchymal pattern of the breast and risk of breast cancer has been reported by Wolfe (1976*a*–*c*) and others, and has been the subject of reviews (Boyd et al, 1984; Oza and Boyd, 1993); carefully conducted studies support an association between parenchymal patterns and breast cancer risk.

In studies that have classified mammographic densities quantitatively, women with many extensive areas of density have consistently been found to have an approximately four- to sixfold increased risk of breast cancer compared with women with no densie areas (Boyd et al, 1982, 1995*a*; Brisson et al, 1982, 1984, 1989; Wolfe et al, 1987; Saftlas et al, 1991; Byrne et al, 1995). The increased risk of breast cancer in women with many areas of dense breast tissue is at least as large as, or larger than, is associated with most other known risk factors for the disease.

Previous studies that have examined the relationship between mammographic densities and other breast cancer risk factors have consistently found an inverse association between body weight and per cent of the breast area occupied by radiologically dense breast tissue (Grove et al, 1979, 1985; Brisson et al, 1982, 1984; Gravelle et al, 1982; Janzon et al, 1982; Whitehead et al, 1985; Boyd and McGuire, 1990; Bartow et al, 1995; Boyd et al, 1995*b*). The purpose of the present paper is to examine this association further. The subjects of this report are premenopausal women, a group in which we have previously noted an association between the Quetelet index and per cent density (Boyd et al, 1995*b*).

### PATIENTS AND METHODS

### General method

Information about risk factors for breast cancer was collected by questionnaire from women without breast cancer but with different degrees of mammographic density, as assessed by radiologists as a percentage of the breast area on a five-point scale. The anthropometric variables of height, weight and skinfold thickness were measured at the time of interview.

### Method of sampling and classification of breast density

*Source of subjects*

The goal of the sampling procedure was to assemble premenopausal women with a wide spectrum of mammographic densities. Subjects aged between 29 and 51 years were identified between 1990 and 1992 from the mammographic units of St. Michael's and Mount Sinai Hospital in Toronto. Women were referred to these units for a variety of reasons, including suspicion of breast disease, the presence of risk factors such as a family history of breast cancer, or for routine examination. Breast density, as provisionally assessed by the radiologist was definitively classified by quantitative methods that are described below.

*Received 6 November 1997*
*Revised 12 March 1998*
*Accepted 18 March 1998*

*Correspondence to:* NF Boyd, Division of Epidemiology and Statistics, Ontario Cancer Institute, 610 University Avenue, Toronto M5G 2M9, Canada

### Method of recruitment

Subjects identified in the manner described above were contacted by a letter that explained the goals and procedures of the study. This was followed by a phone call, during which their eligibility was determined. Subjects were eligible for the study if they were menstruating regularly, were not pregnant or breast feeding, had no previous history of cancer, had not had a hysterectomy or oopherectomy and were not scheduled to have breast surgery. All subjects taking any type of exogenous hormone preparation were excluded. Of the subjects contacted: 65% were eligible for inclusion in the study and, of these, 95% agreed to take part. The rate of participation did not vary according to extent of mammographic density. Subjects who agreed to enter the study were then visited in their homes by the study research assistant during the luteal phase of their menstrual cycle (between days 20 and 24), and the following measurements were made. Subjects were recruited into the present study only after mammograms had been taken, but the phase of the menstrual cycle during which mammograms were obtained was not recorded.

### Anthropometric measures

Each subject was weighed on a balance scale and measured for height. Skinfold thickness in the triceps, subscapular and iliac crest areas was measured using Lange calipers by a research assistant trained and certified by the Department of Athletics and Recreation, University of Toronto, Canada.

### Definitive classification of breast parenchymal pattern

The measurements in the following analysis were made using a randomly selected, craniocaudal (viewing from above, down) mammographic view of one breast from each subject.

Mammograms were digitized and presented for analysis as an array of 675 × 925 pixels (0.0676 $mm^2$ per pixel). The manipulation of images and all calculations of the parameters to be described were performed on a Sun 4/260 workstation (Sun Microsystems, Mountain View, CA,USA). A Megavision 1024xm image processor/display (Megavision, Goleta, CA, USA) was used to present the images to the observer. An interactive density thresholding technique was used with a graphics overlay, in which an observer interactively highlighted a selected pixel value in colour by manipulation of a trackball. The process of measurement is illustrated in Figure 1.

The observer first selected a grey value as a threshold to separate the image of the breast from the background and determined the breast size. A second threshold was then selected to identify the edges of region(s) which are representative of radiographically dense tissue in the image, the sum of which gives the area of density in the breast. The proportion of the total area occupied by the radiographically dense tissue was calculated as the percentage of the entire projected area of the breast, expressed as per cent density. All thresholds were selected by one observer (NFB) who was unaware of any of the characteristics of the subjects. Further details of this method are described elsewhere (Byng et al, 1994). We have found high levels of intra- and inter-reader reliability with the measurement. In the present study, duplicates of a subset of the images were included as a check on reliability, which was found to be high with a test–retest correlation of 0.9 or greater.

### Statistical analysis

Data analysis was carried out using the SAS statistical software package (SAS Institute, 1989). Data were inspected for skewness before analysis and, when necessary, a transformation from the power family was applied. Details of the transformations used are given in the footnotes of the tables of results. The associations between anthropometric variables and mammographic measures were examined using Pearson's correlation coefficients. Multiple linear regression analysis and partial correlations were used to examine the relationship between each of the four measurements obtained from the mammogram and anthropometric variables after adjustment for other anthropometric variables. In addition, all models were controlled for age, age at menarche, parity and a family history of breast cancer. $P$-values ≤ 0.05 were considered to be statistically significant.

**Table 1** Correlation between breast measurements

| | Dense area[a] | Non-dense area[a] | Breast area[a] | Per cent density |
|---|---|---|---|---|
| Dense area | 1.00 | – 0.456[c] (0.0001) | 0.117 (0.0542) | 0.753 (0.0001) |
| Non-dense area | | 1.00 | 0.801 (0.0001) | – 0.886 (0.0001) |
| Breast area | | | 1.00 | – 0.469 (0.0001) |

[a]Measure is transformed using square root. [c]Values shown are Pearson correlation coefficients with $P$-values in brackets.

**Table 2** Correlation between breast measurements and anthropometric variables

| | Dense area[a] | Non-dense area[a] | Breast area[a] | Per cent density |
|---|---|---|---|---|
| Height[b] | 0.002[d] (0.98) | –0.059 (0.33) | –0.022 (0.72) | 0.080 (0.19) |
| Weight[b] | –0.179 (0.003) | 0.601 (0.0001) | 0.584 (0.0001) | –0.464 (0.0001) |
| Quetelet index[c] | –0.191 (0.002) | 0.661 (0.0001) | 0.623 (0.0001) | –0.526 (0.0001) |
| Triceps[a] | –0.214 (0.0004) | 0.539 (0.0001) | 0.449 (0.001) | –0.471 (0.0001) |
| Subscapular[a] | –0.172 (0.004) | 0.585 (0.0001) | 0.529 (0.0001) | –0.483 (0.0001) |
| Suprailiac[a] | –0.226 (0.0002) | 0.583 (0.0001) | 0.527 (0.0001) | –0.485 (0.0001) |
| Body fat[c] | –0.210 (0.0005) | 0.624 (0.0001) | 0.552 (0.0001) | –0.530 (0.0001) |

[a]Square root transformed. [b]Negative inverse transformed. [c]Body fat = triceps + subscapular + suprailiac skinfold thickness; log transformed. [d]Pearson correlation coefficient ($P$-value).

### RESULTS

### Characteristics of subjects

Two hundred and seventy-three subjects were studied. All were premenopausal, with a median age of 43 years (range 29–51), median weight of 64 kg [interquartile range (IQR) 14.5] and median height of 162 cm (IQR 8). Seventy-seven (28%) were nulliparous, 144 (53%) had one or two children and 52 (19%) had

© Cancer Research Campaign 1998

**Table 3** Regression analysis of breast measurements with anthropometric variables: comparison of models[a]

| Model | Dense area[b] | Non-dense area[b] | Breast area[b] | Per cent density |
|---|---|---|---|---|
| Height[c] | 0.075[e] (0.22) | –0.326 (< 0.001) | –0.258 (< 0.001) | 0.266 (< 0.001) |
| Weight[c] | –0.201 (0.001) | 0.652 (< 0.001) | 0.616 (< 0.001) | –0.513 (< 0.001) |
| $R^2$[f] | 7.0% | 46.6% | 42.5% | 30.5% |
| Height | 0.018 (0.77) | –0.227 (< 0.001) | –0.219 (< 0.001) | 0.149 (0.02) |
| Weight | –0.026 (0.67) | 0.315 (< 0.001) | 0.364 (< 0.001) | –0.156 (0.01) |
| Body fat[d] | –0.093 (0.13) | 0.137 (0.03) | 0.017 (0.79) | –0.181 (0.003) |
| $R^2$ | 7.8% | 47.9% | 42.7% | 33.1% |
| Quetelet index[c] | –0.208 (0.001) | 0.660 (< 0.001) | 0.616 (< 0.001) | –0.524 (< 0.001) |
| $R^2$ | 7.2% | 47.4% | 42.5% | 31.3% |
| Quetelet index | –0.039 (0.53) | 0.332 (< 0.001) | 0.363 (< 0.001) | –0.178 (0.004) |
| Body fat | –0.080 (0.20) | 0.110 (0.07) | 0.006 (0.92) | –0.154 (0.01) |
| $R^2$ | 7.9% | 48.4% | 42.7% | 33.2% |

[a]All models contain age, age at menarche, parity and family history. [b]Square root transformed. [c]Negative inverse transformed. [d]Body fat = triceps + subscapular + suprailiac skinfold thickness; log transformed. [e]Partial correlation coefficient controlling for all variables in the model (*P*-value). [f]Total variance explained by regression model.

**Figure 1** Method used to measure mammographic features. White line delineates the edge of the breast and the black line the edge of dense tissue three or more children. Fifty-four (20%) had at least one first-degree relative with breast cancer.

### Distribution of mammographic features

Figure 2 shows the distribution of the mammographic features included in the analyses that follow. The median area of the breast in the mammographic image was 101.0 cm$^2$ (IQR 63), the median area of dense tissue was 39.5 cm$^2$ (IQR 37.9) and the median area of non-dense tissue was 51.2 cm$^2$ (IQR 69.9).

### Relationship between measures of mammographic features

Table 1 shows the Pearson correlation coefficients between the measurements of areas of dense and non-dense tissues, total area and the per cent of the total area of the mammogram occupied by dense tissue. The total area of the breast was strongly correlated with the area of non-dense tissue ($r = 0.801$; $P = 0.0001$) and less strongly with the area of dense tissue ($r = 0.117$; $P = 0.05$). The per cent of the total area occupied by dense tissue was strongly correlated with both dense and non-dense areas, although in opposite directions ($r = 0.753$ and $-0.886$ respectively; $P = 0.0001$ for each), and the areas of dense and non-dense tissue were correlated inversely with each other ($r = -0.456$; $P = 0.0001$).

### Relationship between measures of mammographic features and anthropometric measures

Table 2 shows the Pearson correlation coefficients between the measurements of the mammogram and the anthropometric variables of height, weight, the Quetelet index and skinfold thicknesses measured over triceps, in the subscapular and suprailiac areas. The sum of these three skinfold measures is referred to here as body fat.

Height was not significantly associated with any of the mammographic measures. Weight and the Quetelet index were both strongly (positively) associated with the area of non-dense tissue $r = 0.601$; $P = 0.0001$; and $r = 0.661$; $P = 0.0001$ respectively) and with the total area of the breast ($r = 0.584$; $P = 0.0001$, and $r = 0.623$; $P = 0.0001$), but had a much weaker and negative correlation with the area of dense tissue ($r = -0.179$; $P = 0.003$ and $r = -0.191$; $P = 0.002$). As noted in previous work, weight and the Quetelet index both had a strong negative correlation with the per cent of the breast area occupied by dense tissue ($r = -0.464$; $P = 0.0001$ and $r = -0.526$; $P = 0.0001$)(Boyd et al, 1995*b*).

© *Cancer Research Campaign 1998* 

The association of skinfold thickness with mammographic measures, in general, resembled that of weight. All skinfold measures, and their sum, were strongly and positively correlated with total area and area of non-dense tissue, and negatively with the per cent area of dense tissue. These variables were also negatively, although less strongly, associated with the area of dense tissue.

### Regression analysis and partial correlations of mammographic and anthropometric measures

Because height, weight and the Quetelet index are highly correlated, we examined their separate influences in a series of linear regression analyses (results are shown in Table 3). The partial correlations are given to show the magnitude of the association between each mammographic feature and anthropometric variable, after adjustment for the other variables in the model. All models were controlled for age, age at menarche, parity and family history of breast cancer. The $R^2$ is the variance in the mammographic measure explained by each regression model. The independent variables in each model were a subset of the anthropometric variables shown in the table, plus the variables we controlled for, and each measure obtained from the mammogram was the dependent variable. Because the skinfold thicknesses were all highly correlated with each other, we used only the sum of the three measures.

The anthropometric measures height (negatively), weight (positively) and the Quetelet index (positively) were all independently associated with the area of non-dense tissue. Body fat was statistically significant (positively) only in the model with height and weight. Similar associations were found with the total area of the breast, except that this measures was not independently associated with body fat.

Regression analysis showed that the anthropometric measures, with the variables for which we controlled and depending upon the model, accounted for between 46.6% and 48.4% of the variance in area of non-dense tissue and 42.5–42.7% of the variance in total breast area.

Weight and the Quetelet index were both negatively and significantly associated with the area of dense breast tissue, except when body fat (which is highly correlated with other indices of body size) was included in the model, although the correlation coefficients, in absolute value, were much smaller than was found between these variables and the other mammographic measures. The models containing anthropometric variables and the controlling variables accounted for between 7.0% and 7.9% of the variance in the area of mammographically dense tissue.

Per cent density was significantly and independently associated with height (positively), and with weight, body fat and the Quetelet index (negatively). Models containing these variables and the controlling variables accounted for between 30.5% and 33.2% of the variance in per cent density.

Although height was not significantly correlated with any of the breast measurements in univariate analysis (Table 2), after controlling for weight, as well as age, age at menarche, parity and family history of breast cancer, it became significantly associated in multivariate analysis with total area, area of non-dense tissue (both negatively) and per cent density (positively).

Because the areas of dense and non-dense tissue were inversely correlated with each other, we next examined their influence on the regression analysis of each other with anthropometric measures. The area of dense tissue was included in models given in Table 3 as an additional independent variable in the analysis, with area of non-dense tissue as a dependent variable. Similarly, the non-dense area was included among the independent variables, with area of dense tissue as the dependent variable. The results of these analyses are summarized in Table 4. When the non-dense area was included in the four regression models with dense area as the dependent variable it was highly significant ($P \ll 0.001$), with partial correlations of – 0.449 to – 0.458. In both of the models that contained it, the Quetelet index was significant. Weight was also significant in both models in which it was included. As in the models shown in Table 3, neither height nor body fat was statistically significant in the models shown in Table 4. The variance in the dense area explained by the regression model increased from approximately 7% to 26%, after the inclusion of the area of non-dense tissue as an independent variable.

The regression coefficients for weight in the models in which dense area is the dependent variable are both statistically significant, but are opposite in sign (Tables 3 and 4). The change in sign occurs because weight and non-dense area are correlated, and

**Table 4** Regression analysis: breast measurements and anthropometric variables[a]

| Model | Dense area[b] | Model | Non-dense area[b] |
|---|---|---|---|
| Height[c] | –0.088[e] (0.15) | Height | –0.328 (< 0.001) |
| Weight[c] | 0.159 (0.01) | Weight | 0.645 (< 0.001) |
| Non-dense area | –0.458 (< 0.001) | Dense area | –0.458 (< 0.001) |
| $R^2$[f] | 26.5% | $R^2$ | 57.8% |
| Height | –0.095 (0.12) | Height | –0.244 (< 0.001) |
| Weight | 0.131 (0.03) | Weight | 0.338 (< 0.001) |
| Body fat[d] | –0.035 (0.58) | Body fat | 0.106 (0.09) |
| Non-dense area | –0.451 (< 0.001) | Dense area | –0.451 (< 0.001) |
| $R^2$ | 26.6% | $R^2$ | 58.5% |
| Quetelet index[c] | 0.155 (0.01) | Quetelet index | 0.651 (< 0.001) |
| Non-dense area | –0.454 (< 0.001) | Dense area | –0.454 (< 0.001) |
| $R^2$ | 26.3% | $R^2$ | 58.3% |
| Quetelet index | 0.126 (0.04) | Quetelet index | 0.351 (< 0.001) |
| Body fat | –0.034 (0.59) | Body fat | 0.083 (0.18) |
| Non-dense area | –0.449 (<0.001) | Dense area | –0.449 (< 0.001) |
| $R^2$ | 26.5% | $R^2$ | 58.8% |

[a]All models contain age, age at menarche, parity and family history. [b]Square root transformed. [c]Negative inverse transformed. [d]Body fat = triceps + subscapular + suprailiac skinfold thickness; log transformed. [e]Partial correlation coefficient controlling for all variables in the model ($P$-value). [f]Total variance explained by regression model.

© Cancer Research Campaign 1998

We also found a negative relationship between the dense and non-dense areas in the mammogram. Because the total breast area is composed of only dense and non-dense tissue, there must be a relationship between the per cent of the total area occupied by these types of tissue. However, the actual areas of dense and non-dense tissue might vary independently, and there is no reason to suppose they should be correlated. However, variations in the non-dense area accounted for 21% of the variance in the area of dense tissue, and the negative association of the areas of these two tissue types suggests a common underlying mechanism related to their formation.

As both the dense and non-dense area measurements involved the same measurement process, we explored the possibility that the observed dense/non-dense correlation was explained by correlated measurement errors for the two variables. A sample of 30 subjects had replicate measurements of these variables from four different observers. These data were used to model the measurement error. When measurement error was adjusted for in this sample, the observed dense/non-dense correlation changed only by 0.01. As this model for measurement error probably overestimates the error for a specific observer, we conclude that correlated errors cannot account for our results.

Radiologically dense breast tissue is composed of fibrous stroma and epithelium, and non-dense tissue is composed of mainly fat. Several potential mechanisms exist to explain a quantitative relationship between the tissues responsible for the dense and non-dense radiological components of the breast. Adipocytes in the breast develop from preadipocytes that are part of the breast stroma and have the morphology of fibroblasts (Ailhaud et al, 1992). This terminal differentiation, which is associated with the accumulation of fat in adipocytes, may be associated with a reduction in the area of radiologically dense tissue in the mammogram and an increase in the area of radiolucent tissue. A number of interactions have been described between adipocytes and mammary epithelium. In vitro experiments show that adipocytes exert an influence on mammary epithelial cell proliferation, probably through an effect on extracellular components, and also promote epithelial cell differentiation (Roncari and Hamilton, 1993; Xu and Bjorntorp, 1987). Adipocytes, as well as epithelial and other stromal cells in the breast, are influenced by sex hormones. For example, the activity of lipoprotein lipase in adipocytes is controlled by progesterone, which also is thought to play a role in proliferation of epithelial cells in the breast (Xu and Bjorntorp, 1987).

These results indicate that the relationship of dense and non-dense tissue areas in the mammogram should be examined separately in relation to other risk factors for breast cancer, and their associations with risk of the disease determined. Combining dense and non-dense areas into a single index of per cent dense tissue, as has been done to date in studies of breast cancer risk, may not be the optimal way of treating this information and alternatives should be examined.

### REFERENCES

Ailhaud G, Grimaldi P and Negrel R (1992) Cellular and molecular aspects of adipose tissue development. *Annu Rev Nutr* **12**: 207–233

Bartow SA, Pathak DR, Mettler FA, Key CR and Pike MC (1995) Breast mammographic pattern: a concatenation of confounding and breast cancer risk factors. *Am J Epidemiol* **142**: 813–819

Boyd NF and McGuire V (1990) Evidence of association between plasma high density lipoprotein cholesterol and risk factors for breast cancer. *J Natl Cancer Inst* **82**: 460–468

Boyd NF, O'Sullivan B, Campbell JE, Fishell E, Simor I, Cooke G and Germanson T (1982) Mammographic signs as risk factors for breast cancer. *Br J Cancer* **45**: 185–193

Boyd NF, O'Sullivan B, Fishell E, Simor I and Cooke G (1984) Mammographic patterns and breast cancer risk: methodologic standards and contradictory results. *J Natl Cancer Inst* **72**: 1253–1259

Boyd NF, Byng JW, Jong RA, Fishell EK, Little LE, Miller AB, Lockwood GA, Tritchler DL and Yaffe MJ (1995*a*) Quantitative classification of mammographic densities and breast cancer risk: results from the Canadian National Breast Screening Study. *J Natl Cancer Inst* **87**: 670–675

Boyd NF, Connelly P, Byng J, Yaffe M, Draper H, Little L, Jones D, Martin LJ, Lockwood G and Tritchler D (1995*b*) Plasma lipids, lipoproteins, and mammographic densities. *Cancer Epidemiol Biomarkers Prev* **4**: 727–733

Brisson J, Merletti F and Sadowsky NL (1982*a*) Mammographic features of the breast and breast cancer risk. *Am J Epidemiol* **115**: 428–437

Brisson J, Sadowski NL, Twaddle JA, Morrison AS, Cole P and Merletti F (1982*b*) The relation of mammographic features of the breast to breast cancer risk factors. *Am J Epidemiol* **115**: 438–443

Brisson J, Morrison AS and Kopans DB (1984) Height and weight, mammographic features of breast tissue, and breast cancer risk. *Am J Epidemiol* **119**: 371–381

Brisson J, Verreault R, Morrison A, Tennina S and Meyer F (1989) Diet, mammographic features of breast tissue, and breast cancer risk. *Am J Epidemiol* **130**: 14–24

Byng JW, Boyd NF, Fishell E, Jong RA and Yaffe MJ (1994) The quantitative analysis of mammographic densities. *Phys Med Biol* **39**: 1629–1638

Byrne C, Schairer C, Wolfe J, Parekh N, Salane M, Brinton LA, Hoover R and Haile R (1995) Mammographic features and breast cancer risk: effects with time, age, and menopause status. *J Natl Cancer Inst* **87**: 1622–1629

Carlile T, Kopecky KJ, Thompson DJ, Whitehead JR, Gilbert FI, Present AJ, Threatt BA, Krook P and Hadaway E (1985) Breast cancer prediction and the Wolfe classification of mammograms. *JAMA* **254**: 1050–1053

Gravelle IH, Bulstrode JC and Bulbrook RD (1982) The relation between radiological patterns of the breast and body weight and height. *Br J Radiol* **55**: 23–25

Grove JS, Goodman MJ, Gilbert FI and Clyde D (1979) Factors associated with breast structures in breast cancer patients. *Cancer* **43**: 1895–1899

Grove JS, Goodman MJ and Gilbert F (1985) Factors associated with mammographic pattern. *Br J Radiol* **58**: 21–25

Hunter DJ and Willett WC (1993) Diet, body size, and breast cancer. *Epidemiol Rev* **15**: 110–132

Ingleby H and Gerson-Cohen J (1960) *Comparative Anatomy, Pathology and Roentgenology of the Breast*. University of Philadelphia Press: Philadelphia

Janzon L, Andersson I and Petersson H (1982) Mammographic patterns as indicators of risk of breast cancer. *Radiology* **143**: 417–419

Oza AM and Boyd NF (1993) Mammographic parenchymal patterns: a marker of breast cancer risk. *Epidemiol Rev* **15**: 196–208

Roncari DAK and Hamilton BS (1993) Cellular and molecular factors in adipose tissue growth and obesity. *Adv Exp Med Biol* **334**: 269–277

Saftlas AF, Hoover RN, Brinton LA, Szklo M, Olson DR, Salane M and Wolfe JN (1991) Mammographic densities and risk of breast cancer. *Cancer* **67**: 2833–2838

SAS Institute inc (1989) *SAS/STAT User's Guide, Version 6*. SAS Institute: Cary, NC

Whitehead J, Carlile T, Kopecky KJ, Thompson DJ, Gilbert FIJR, Present AJ, Threatt BA, Krook P and Hadaway E (1985) The relationship between Wolfe's classification of mammograms, accepted breast cancer risk factors, and the incidence of breast cancer. *Am J Epidemiol* **122**: 994–1006

Willett WC, Browne ML, Bain C, Lipnick RJ, Stampfer MJ, Rosner B, Colditz GA, Hennekens CH and Speizer FE (1985) Relative weight and risk of breast cancer among premenopausal women. *Am J Epidemiol* **122**: 731–740

Wolfe JN (1976*a*) Breast parenchymal patterns and their changes with age. *Radiology* **121**: 545–552

Wolfe JN (1976*b*) Breast patterns as an index of risk for developing breast cancer. *Am J Roentgenol* **126**: 1130–1139

Wolfe JN (1976*c*) Risk for breast cancer development determined by mammographic parenchymal pattern. *Cancer* **37**: 2486–2492

Wolfe JN, Saftlas AF and Salane M (1987) Mammographic parenchymal patterns and quantitative evaluation of mammographic densities: a case–control study. *Am J Roentgenol* **148**: 1087–1092

Xu X and Bjorntorp P (1987) Effects of sex steroid hormones on differentiation of adipose precursor cells in primary culture. *Exp Cell Res* **173**: 311–321

© Cancer Research Campaign 1998